# Multi-Criteria Evaluation Model of Management for Weaned Piglets and Its Relations with Farm Performance and Veterinary Medicine Consumption

**DOI:** 10.3390/ani13223508

**Published:** 2023-11-14

**Authors:** Santos Sanz-Fernández, Cipriano Díaz-Gaona, Carmen Borge, Raquel Quintanilla, Vicente Rodríguez-Estévez

**Affiliations:** 1 Department of Animal Production, UIC Zoonoses and Emerging Diseases (ENZOEM), Faculty of Veterinary Medicine, International Agrifood Campus of Excellence (ceiA3), University of Córdoba, Campus de Rabanales, 14071 Córdoba, Spain; 2 Department of Animal Health, Faculty of Veterinary Medicine, International Agrifood Campus of Excellence (ceiA3), University of Córdoba, Campus de Rabanales, 14071 Córdoba, Spain; 3 Animal Breeding and Genetics Program, Institute of Agrifood Research and Technology (IRTA), 08140 Caldes de Montbui, Spain

**Keywords:** post-weaning, veterinary hygiene, handling, husbandry, quick scan, calculator, animal welfare

## Abstract

**Simple Summary:**

Weaning is a key moment in the pig’s life that is often fraught with stress and immunosuppression. Poor handling of piglets during weaning compromises their health and performance, potentially resulting in increased expenditure on veterinary medicines. Therefore, the aim of this work is to design a quick scan calculator, a multi-criteria assessment tool, built upon ten indices based on hygienic and handling measures. These indices encompass pre-weaning handling, batch management, biosecurity, water and feed management, health programs, stockmen training, temperature, ventilation, and floor conditions and density. Each index receives a maximum score of 10, and the cumulative score reflects the degree of adequacy of on-farm management, with a perfect score being 100. Field testing across 23 farms unveiled the highest scores for floor conditions and density, along with pre-weaning handling and health programs. Conversely, temperature, ventilation, water management, and stockmen training scored lower. The average farm score stood at 56.12 out of 100. Importantly, the calculator’s score correlated significantly with key post-weaning piglet health and productivity parameters. By focusing on the indices with lower scores, farms can improve management, hygiene practices, and preventive measures, ultimately reducing medication use and enhancing overall piglet welfare.

**Abstract:**

Weaned piglets, being immature, demand careful handling to mitigate post-weaning stress in order to avoid immunosuppression and the use of antimicrobials to palliate the effects of disease outbreaks due to poor management. The objective of this work is to design a quick scan calculator or multi-criteria evaluation model of management for weaned piglets, founded on 10 critical indices covering post-weaning management aspects based on hygienic measures and management of facilities and animals. These include pre-weaning handling, batch management, biosecurity, water management, feed management, health program, stockmen training, temperature, ventilation, and floor conditions and density to relate handling and hygiene practices with farm performance and the consumption of veterinary medication. Each index carries a maximum score of ten, with evaluations derived from different management factors that make up each index (from three to eight factors were evaluated per index). Their cumulative score reflects the degree of adequacy of on-farm management. Therefore, a perfectly managed farm would achieve 100 points. The calculator underwent testing on 23 intensive farms with a total population of close to 16,000 sows and more than 400,000 weaned piglets, revealing the highest mean scores in floor conditions and density (8.03 out of 10) and pre-weaning handling and health programs (6.87 and 6.28, respectively). Conversely, the lowest scores corresponded to temperature, ventilation, water management, and stockmen training (4.08, 4.32, 4.81, and 4.93, respectively). The assessed farms averaged a global score of 56.12 out of 100 (from 37.65 to 76.76). The calculator’s global score correlated with key post-weaning productivity and piglet health indicators, such as the feed conversion ratio, mortality rate, and piglet production cost, with r values of −0.442, −0.437, and −0.435, respectively (*p* < 0.05). Additionally, it negatively correlated with medication costs per piglet (r = −0.414; *p* < 0.05) and positively with annual farm productivity (r = 0.592; *p* < 0.01). To enhance management, hygiene, and prevention, farms should prioritize addressing indices with the lowest scores, thereby reducing medication consumption and enhancing productivity and health outcomes. Additionally, this quick scan calculator can be used for benchmarking purposes.

## 1. Introduction

Weaning is the most critical and stressful period in the productive life of pigs. The start of the post-weaning phase is characterized by significant social and physiological changes because piglets are separated from the sow, transitioned to solid feed, and placed in new facilities and social groups [1,2]. In the context of existing commercial weaning methods, these changes can lead to stress and increase susceptibility to disease, morbidity, mortality, suboptimal growth performance, and costs [3,4,5]. For instance, immunosuppression may increase susceptibility to gastrointestinal diseases, which requires treatment or management to reduce this effect. Consequently, psychosocial stress is a major factor driving gastrointestinal tract pathophysiology and disease susceptibility [6]. These social and physiological stresses are not easily avoidable but they can be alleviated through proper management and hygiene, thereby enhancing animal welfare and achieving improved productivity and health outcomes [2,7].

Until recently, part of the problems derived from stress and poor hygiene and management conditions on farms were mitigated through preventive therapy measures, basically including the use of antimicrobials in feed or water, such as colistin or zinc oxide. However, the increased concern regarding bacterial resistance and social demands [8] has limited the possibilities of antibiotic prevention. In this regard, the European Food Safety Authority (EFSA) and the European Medicines Agency (EMA) published a set of measures to reduce the need for antimicrobial treatments in animal breeding in the EU and the resulting impacts on antimicrobial resistance [9].

Currently, there are protocols for assessing animal welfare, such as “Welfare Quality” [10], as well as studies proposing management measures and factors to consider in order to improve the productivity and health outcomes of farms [11,12,13,14] or studies evaluating measures to reduce the use of antimicrobials [5,15,16,17,18,19,20,21]. In this sense, some of the factors that improve productivity include the number of pigs per pen and the type of feeders [14], as well as biosecurity elements, such as the implementation of an all-in, all-out protocol or the practice of changing clothes and boots between different groups of pigs to prevent infectious diseases [12]. However, there is a need for indices and assessment schemes for overall management and hygiene on farms, which include aspects related to biosecurity, production flow, batch dynamics, a reduction in antimicrobial use, and animal welfare from the handling of facilities perspective.

Therefore, the objective of this study was to design a quick scan calculator based on handling and hygiene indices to evaluate the hygienic sanitary conditions and husbandry and management practices during the post-weaning phase. Additionally, to validate this calculator, the scores obtained for the different indices in a set of farms are correlated with their productivity, health, and cost results. Finally, these scores could be used for on-farm self-monitoring and benchmarking purposes. Therefore, if poor management of piglets during weaning compromises their health and performance, it may lead to increased spending on veterinary medicines. It is expected that farms with improved handling and hygiene practices during the post-weaning phase have higher productivity, better health outcomes, and reduced costs compared to farms with suboptimal practices.

## 2. Materials and Methods

### 2.1. Data Source

The study was performed with a sample of 23 intensive commercial farms, located in the southern region of Spain, with a total population close to 16,000 sows and total yearly production slightly exceeding 400,000 weaned piglets. Those farms amounted to a total of 242 post-weaning rooms, where every facility and piece of equipment were evaluated, including both pre-weaning and post-weaning handlings. When the farms were visited, facilities and management practices affecting 6 batches simultaneously were evaluated (since, on average, the post-weaning phase lasted 6 weeks in the 23 farms); therefore, 11.5% of the yearly batches would have been assessed in each farm because these farms produced weekly batches. This prospective study involved the collection of data for the calculator directly on the farms. Following the farm evaluations, information regarding productive and health parameters was requested. This approach was adopted to prevent any pre-existing knowledge, which could influence the objectivity of the farm assessment.

### 2.2. Methodology for Evaluating the Indices and Factors of the Quick Scan Handling and Hygiene Calculator

Data collection and evaluation of the handling, hygiene, health, and facility conditions of each farm were carried out through farm visits and interviews with farmers based on a questionnaire that comprised all those topics. That information was registered in an Excel© spreadsheet designed as a quick scan calculator, which includes information on 10 indices to evaluate different management factors (Table 1). This calculator was designed without any prior knowledge of the evaluated farms. These indices were designed based on pig production standards [22] and studies on animal welfare, husbandry, handling, and hygiene [2,7,23,24,25]. Each index was considered a limiting factor that, on its own, could trigger any health or production problem for any farm, and was scored up to 10 points. The last three indices (temperature management, room ventilation management, and room floor type and density) were evaluated individually for each post-weaning room (Table 1), so the farm’s score for these indices was the mean score of all the post-weaning rooms within the corresponding farm.

The score for each index comes from the partial scores assigned to specific management factors (from 3 to 8 factors per index; Table 1) based on the importance given to them by different authors [2,7,22,23,24,25]. The weight given to each factor, or partial score within an index (Table S1), was agreed upon by the authors and contrasted with expert swine veterinary consultants. The assessment of factors was resolved using a checklist, aiming for the simplest possible answer, which could be either the presence or absence of good practice or several categories of response. The highest score (10 out of 10) for each index corresponded to perfect handling, hygiene, and facilities, and the score obtained for each index depends on the sum of the scores for each evaluated management factor. A farm with perfect management and hygiene would achieve a total score of 100 points.

### 2.3. Evaluation of the Productive and Health Parameters of the Farms

The mean productive and health results of each farm were evaluated over a one-year period to compare these with the scores obtained in the calculator’s indices. The collected data included the mean number of reproductive sows in the farm; annual productivity (piglets weaned per sow per year, PWSY); pre-weaning piglet mortality rate (%); piglet weight during weaning (kg); cost of weaned piglet (EUR), including costs related to sows (replacement cost, feed, mating, etc.); post-weaning average daily gain (ADG) (g/d); post-weaning feed conversion ratio (FCR); post-weaning medication costs (EUR) per weaner (considering all veterinary drugs, including antimicrobials and any commercial product requiring veterinary prescription); post-weaning piglet mortality rate (%); post-weaning total cost per piglet (feed, husbandry, and medication) (EUR/piglet); and post-weaning cost per kg of live weight (LW) produced (EUR/kg LW).

### 2.4. Statistical Analysis

IBM SPSS^®^ Statistics version 22 software was used to perform the statistical analyses. The descriptive statistics were calculated for productive and health parameters, as well as for the scores of the calculator indices of all the farms. Subsequently, Pearson correlation coefficients between these scores and the productive and health results of the farms were calculated to estimate their possible linear association (asterisks in the tables indicate significant correlations).

Additionally, the farms were grouped into quartiles based on PWSY and medication costs per piglet during the post-weaning phase. Parametric tests were applied once the normality of the variables involved was verified. An ANOVA test was conducted, followed by a Student–Newman–Keuls test, to analyze the distribution of differences among the quartile groups in relation to the overall score of the quick scan handling and hygiene calculator and the productive and health outcomes. Different superscripts (^a b^) have been used to indicate significant differences (*p* < 0.05) between groups.

## 3. Results

The descriptive statistics of the productive and health results of the farms studied are shown in Table 2. The mean annual productivity was 24.60 PWSY, with 10.28% pre-weaning piglet mortality, 3.46% post-weaning piglet mortality, post-weaning ADG of 311 g/d, and post-weaning FCR of 1.76. Thus, in this phase, the mean production cost was EUR 45.14/piglet, including EUR 5.69/piglet with veterinary medication.

### 3.1. Evaluation of the Quick Scan Handling and Hygiene Calculator Indices

Table 3 shows the descriptive statistics of the evaluated farms for the 10 veterinary management and hygiene indices proposed. On average, the farms obtained a score of 56.12 out of 100. Generally, the highest scores corresponded to floor type and density in post-weaning rooms, pre-weaning handling, and health program indices, with means of 8.03, 6.87, and 6.28 out of 10, respectively (refer to Table 1 for a detailed description of the indices). On the other hand, the lowest scores were for temperature, ventilation, water management, and farm stockmen training indices, with means of 4.08, 4.32, 4.81, and 4.93 out of 10, respectively. In this sense, it is worth noting that only 4.3% of the farms have localized heating (with thermal floor or plate) in post-weaning facilities, and only 13% of farms implement a minimum (5–10%) ventilation program in these nursery rooms. Additionally, none of the farms offer performance-based incentives to farm stockmen.

**Figure 1:**
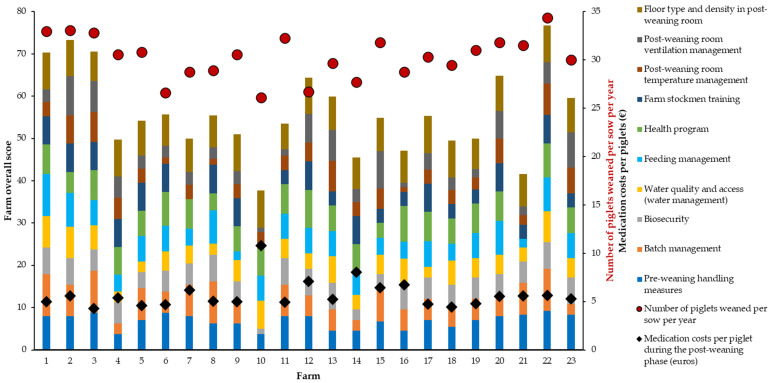
Veterinary management and hygiene indices scores per farm obtained with the quick scan handling and hygiene calculator, number of piglets weaned per sow per year, and medication costs per piglet during the post-weaning phase (N = 23).

Additionally, Figure 1 shows the scores of the veterinary management and hygiene indices, PWSY performance, and medication costs per piglet during the post-weaning phase for each farm. Eight farms (34.4%) obtained overall scores of less than 50 out of 100 using the quick scan handling and hygiene calculator, which is clearly a low score, meaning bad handling.

### 3.2. Correlation between Handling and Hygiene Scores and Productive Parameters

The Pearson correlations between the scores of the quick scan handling and hygiene calculator indices and the productive and health parameters of the farms are shown in Table 4. In general, the overall score of the calculator is significantly correlated with the main productive parameters during the post-weaning phase: FCR, mortality rate, and production costs per piglet (r = −0.442, r = −0.437, and r = −0.435, respectively; *p* < 0.05), and there is also a negative correlation with medication costs per piglet during the post-weaning phase (r = −0.414, *p* < 0.05). Finally, the overall score of the farms is highly significantly correlated with PWSY (r = 0.592, *p* < 0.01).

On the other hand, when evaluating the indices of the calculator independently, it is found that pre-weaning handling is positively correlated with PWSY and negatively correlated with the cost per kg LW during the post-weaning phase (r = 0.531 and r = −0.561, respectively; *p* < 0.01). Similarly, this index is also negatively correlated with the medication costs per piglet (r = −0.473; *p* < 0.05). Batch management and biosecurity are positively correlated with PWSY (r = 0.679 and r = 0.547, respectively; *p* < 0.01) and negatively correlated with medication costs per piglet (r = −0.536 and r = −0.619; *p* < 0.01). Furthermore, batch management is also negatively correlated with the cost per kg LW during post-weaning (r = −0.557, *p* < 0.01). Finally, the score obtained in farm stockmen training is negatively correlated with the medication costs per piglet and the piglet mortality rate during post-weaning (r = −0.432 and r = −0.436, respectively; *p* < 0.05).

### 3.3. Farm Groups of Productivity

Farms were grouped into quartiles according to their annual productivity (PWSY) (Table 5). The quartile of farms with the highest productivity (mean of 27.73 PWSY) obtained the highest mean overall score of the quick scan handling and hygiene calculator (mean of 68.23 out of 100) (*p* < 0.05). Similarly, this most productive group of farms also had better productive and health results during weaning, with a lower mortality rate during lactation (7.42%) and a lower production cost per piglet during weaning (EUR 22.31).

Likewise, these most productive farms also achieved better productive and health results during the post-weaning phase, particularly in comparison to the quartile of farms with lower annual productivity (20.20 PWSY), showing significant differences (*p* < 0.05) between Q1 and Q4 groups for medication costs (EUR 5.16 vs. EUR 7.25), post-weaning piglet mortality rate (2.91% vs. 4.93%), piglet production cost (EUR 42.12 vs. EUR 49.56), and cost per kg LW (EUR 2.14 vs. EUR 2.57).

When comparing the groups of farms based on post-weaning medication costs per piglet (Table 6), it is observed that the lowest overall score of the quick scan handling and hygiene calculator corresponded to the quartile of farms with the highest medication costs (mean of EUR 7.54), although differences between groups were not significant. On the other hand, farms with the highest medication costs (Q1) also exhibited the lowest annual productivity mean (20.78 PWSY) and the highest production cost per weaned piglet (EUR 28.02). Furthermore, this same quartile of farms also had the poorest productive and health results during post-weaning (*p* < 0.05), with 4.74% mortality, EUR 50.31 per weaner, and EUR 2.59/kg LW.

## 4. Discussion

The evaluated farms, with a mean annual productivity of 24.60 PWSY, have lower performance compared to the current Spanish mean (29.38 PWSY) [26]. Among the farms evaluated, only the most productive one, which achieved 28.72 PWSY, came close to these results. The studied farms were low-tech farms with poorer productive results; therefore, they have much room for improvement, highlighting the need to assess their husbandry, handling, and hygiene practices to improve these results. In this regard, the scores of the farms for the 10 indices of the quick scan handling and hygiene calculator (with a mean overall score of 56.12 out of 100) indicate that although 65.2% of the farms scored above 50 points, it cannot be ruled out that these farms may still have management and hygiene errors and shortcomings. Furthermore, in accordance with Liebig’s Law of the Minimum (also known as Liebig’s barrel), indices with low scores, such as biosecurity, farm stockmen training, post-weaning room temperature, or ventilation management, could act as limiting factors in ensuring proper handling and hygiene, which are essential for achieving good performance and health during post-weaning in the studied farms.

When evaluating these indices, the pre-weaning handling, with a mean score of 6.87 out of 10, is one of the indices with a better overall score. Thus, proper handling during lactation is crucial for the successful start of the post-weaning phase to ensure proper colostrum intake in the first few hours after farrowing [27,28,29]. Early access to water and feed enables lactating piglets to start consuming these as early as possible. It is important to offer feed frequently (at least twice a day) in a clean feeder to stimulate solid feed intake from the beginning of their lives [2]. However, 21.7% of the farms obtained a score of <5 points in this index, compromising the good start of weaning.

The mean number of post-weaning rooms per farm was 10.52. Nevertheless, 25% of the farms had an average number of post-weaning rooms below seven. This poses a challenge for implementing the all-in all-out veterinary hygiene principle for post-weaning batch management. Specifically, in cases where piglet production occurs in weekly batches, adhering to this hygiene principle requires 7 weeks of occupation with pigs [7] and 8 weeks of rotation to have time for cleaning and disinfection. Consequently, batch management had a mean score of 5.98 points, including crucial aspects, such as attention to smaller piglets or segregation by weight in pens to reduce hierarchical competition, which leads to significant differences in water and feed consumption and even pathology morbidity and mortality after weaning [30]. Segregating piglets by body weight is a common management strategy to reduce weight variability and facilitate pigs’ handling [31]. Therefore, in this study, segregation by weight has been considered as a best practice. However, the crucial factor lies in having a clear segregation criterion to minimize growth disparities during the post-weaning period. This could also be extended to piglet segregation based on litter or gender. López-Vergé et al. [32] pointed out that despite initial efforts to segregate piglets by weight at the beginning of the post-weaning phase, the coefficient of variation in piglet weight tends to rise, reaching levels comparable to piglets segregated by litter, due to an early socialization strategy.

Overall, the biosecurity mean scores were low (5.11 points), with 17.4% of the farms having values well below five. This low score is due to the lack of basic measures such as footbaths, isolation or sickbay facilities, proper cleaning and disinfection, and proximity to other farms or roads. These hygiene measures are essential to prevent the entry and spread of diseases on the farm. Currently, there are good internal and external biosecurity evaluation systems described by various authors [12,33,34,35], who demonstrated the importance of farm biosecurity measures in reducing antimicrobial consumption and improving production outcomes.

Regarding water management, 60.9% of the farms obtained a mean score below five points due to poor water quality, lack of chlorination or purification treatment, and the absence of good management practices. Drinking water must be clean, fresh, colorless, and free from microorganisms [36], and its good quality is indispensable during post-weaning [7]. In the feed management index, 30.4% of the farms score less than five points due to management errors, such as the lack of feeders with water or mash feed for piglets at early weaning, as well as the absence of rehydration measures during the first days. Implementing these management measures promotes early water and feed consumption, resulting in a better start and intestinal health of the piglet [37]. Additionally, a decrease in feed intake during the first week after weaning is strongly correlated with the risk of disease occurrence during this phase [38,39]. Furthermore, Raasch et al. [19] indicated that improving the quality or composition of feed or water is the most commonly implemented intervention on farms as an alternative to reducing antimicrobial use and improving consumption. In this sense, the design of the feeder also influences feeding behavior habits [40]; thus, O’Connell et al. [41] indicated that the dry multi-space feeder could be the most optimal feeder for weaned piglets in terms of performance and animal welfare.

The training of farm stockmen also obtained a low mean score (4.93 out of 10). However, this index is crucial for the proper functioning of any farm because trained and skilled stockmen know good animal handling and consider all possible negative and positive influencing factors [42]. Hence, it is important to provide clear instructions to workers, including critical points to check and a daily routine that facilitates their work [37]. Magallón et al. [43] emphasize the importance and effect of theoretical and practical training of farm workers on short- or medium-term productivity and indicate that by means of training, it is possible to increase the number of weaned piglets per litter by almost one in just one year. In addition, Spoolder and Ruis [44] pointed out some of the most important attributes a professional stockman needs: a solid technical understanding of what weaned piglets need; a sharp ability to interpret the signals animals give regarding their health and welfare status; and the ability to take action based on that information. Furthermore, proficiency in skills, such as understanding post-weaning targets and associating animal performance with their handling and health, is crucial, as they directly affect farm profitability.

The scores for the temperature and ventilation indices were also low, with only 30.4% and 39.1% of farms meeting the temperature and ventilation criteria, respectively. However, both environmental indices are crucial for maintaining productivity and reducing piglet stress and respiratory diseases during weaning [45,46]. Thus, post-weaning rooms should be heated to 28–30 °C for piglet entry, avoiding daily fluctuations and gradually reducing the temperature by 1–2 °C each week until reaching 22 °C at the end of the phase [7]. Additionally, room temperature also depends on the flooring and stocking density and recommends plastic slatted floors, which have lower heat loss compared to concrete floors [47]. On the other hand, in cold weather, it is important not to compromise ventilation in an attempt to maintain temperature. Always ensure a minimum ventilation flow while avoiding exposing the animals to cold air, and maintain a maximum air velocity of 0.15 m/s at the piglet height [7,48]. The type of flooring also affects air quality, increasing ammonia concentration with partial slats compared to total slatted pens [49]. In general, the goal of ventilation is to renew the volume of air in the weaning rooms, prevent respiratory pathologies, regulate temperature, eliminate harmful gases, and ensure the necessary supply of oxygen [7]. In this regard, there are many studies suggesting strategies and better facilities to improve the air quality of farms [7,47,49,50,51]. However, caution must be taken with newly weaned piglets, as they are highly susceptible to low temperatures and high air speeds [47].

Additionally, the Pearson correlations validate the usefulness of the quick scan handling and hygiene calculator, as the overall score of the calculator correlates significantly (*p* < 0.05) with the main productivity parameters of piglets during the post-weaning phase (FCR, mortality rate, and production cost). Thus, farms that achieved a higher score in the overall evaluation of management and hygiene indices had lower production costs and lower mortalities during the post-weaning phase. These results are in agreement with other authors [52,53] who linked the influence of different management factors to the productivity parameters of farms. Additionally, there is also a negative correlation with the medication costs per piglet during the post-weaning phase (*p* < 0.05), indicating that farms with higher handling scores have lower medication consumption. Therefore, the best way to reduce the consumption of antimicrobials is to guarantee good handling and hygiene to reduce the occurrence of diseases [17].

Furthermore, indices such as biosecurity, batch management, and pre-weaning handling correlate with post-weaning productivity and medication expenses. Thus, Postma et al. [35] evaluated the relationship between biosecurity, productive parameters, and antimicrobial use in four EU countries, concluding that good management practices and biosecurity measures are factors that impact antimicrobial consumption and productivity parameters.

On the other hand, the indices of floor type and density, ventilation and temperature of the post-weaning rooms, and water management do not show significant correlations with the studied productivity and health parameters [47], indicating that these management and hygiene indices influence the mortality rate and the productivity during post-weaning. Additionally, some authors propose other management measures or factors [11,13,14], such as the age of the building, to evaluate the air quality and its bacterial load or suggest fewer piglets per pen, which could be included in future versions of this quick scan calculator.

When comparing farms according to the quartiles of annual productivity and cost of medications per piglet, it is observed that farms with higher annual productivity also achieve better productivity and health outcomes during the post-weaning phase of the piglets. These results align with a study conducted by Pierozan et al. [54], where they indicate a parallelism between productivity increase and the improvement of management practices, biosecurity measures, and handling techniques. Although PWSY is linked to the pre-weaning phase, annual productivity is the parameter that best reflects farm efficiency [55], so it was considered relevant to compare farms according to their level of efficiency based on this parameter. Hence, these results demonstrated that farms achieving good results during weaning also perform well in the post-weaning phase, with lower post-weaning mortality, reduced medication expenses, and lower piglet cost.

On the other hand, the group of farms with higher costs of medication per piglet had poorer productivity and health parameters. Although the use of antimicrobials in farms is currently being reduced, the relationship between the use of antimicrobial drugs to improve farm production has been recognized for decades [56,57,58], especially in swine production [59,60,61]. Therefore, efforts should continue to be made for their control worldwide, as a recent study confirms an association between the use of antimicrobials on farms and their resistance in humans [62]. Additionally, the results obtained are in accordance with Diana et al. [5], who indicate that improvement in management practices can reduce antimicrobial use without significantly affecting the productivity and health outcomes of the farms.

Finally, the possible margin of error in the design of this quick scan calculator would be compensated by the high number of indices evaluated (10), their low contribution to the total score (maximum of 10 out 100 per index), and how evenly that contribution is distributed within different management factors (from four to eight per index). Additionally, this quick scan calculator does not require animal-based measures, so it is quick to use and avoids animal manipulations. Overall, this quick scan handling and hygiene calculator has been designed to assess routine practices within a farm. However, its functionality may be constrained under conditions of severe disease. For example, in the event of a disease outbreak, both production and health parameters would be profoundly affected, even with appropriate piglet and facility management. Additionally, some indices are overly simplistic: the health program index only considers two PRRS status situations; the assessment of biosecurity does not differentiate between internal and external biosecurity; and the evaluation of water quality does not take into account the results of the periodic analyses (it only considers the fact of analyzing water as a first step for its hygiene). To address these issues, a more comprehensive version of the calculator could be developed; however, this would necessitate a significantly larger questionnaire, compromising its aim of being a quick and user-friendly tool for farmers and technicians. For a more complete evaluation of some indices, there are other questionnaires; for example, Pitkin et al. [63] for PRRS, Pritchard et al. [64] for biosecurity, Edwards and Crabb [65] for water management, or the wean to finish guidelines of PIC [66] for ventilation and other issues.

## 5. Conclusions

The proposed quick scan handling and hygiene calculator allows us to evaluate a wide range of management and hygiene aspects of farms that influence their productivity, costs, health, and pharmaceutical consumption. Improving handling and prevention are the basis and key steps toward reducing the use of antimicrobial drugs. With that aim, each farm should start by addressing its indices with the lowest scores. Likewise, for an effective improvement in management, clear instructions should be provided to farm stockmen, promoting their training.

This calculator can be useful for benchmarking purposes between farms and within the same farm to analyze progress and improvements and avoid animal manipulations to evaluate animal welfare. Additionally, although the factors comprising each index have a limited impact on the final score, they could be corrected after testing the calculator on a larger number of farms with benchmarking methodology. With such correction or calibration, the correlations between the calculator’s index scores and productivity and health parameters should increase, bringing the calculator closer to an accurate assessment of farm reality and improving its utility; however, the aim of this first proposal of the calculator was not to adjust its factor scores to fit those correlations.

## Figures and Tables

**Table 1:** Indices and management factors of the quick scan handling and hygiene calculator.

Indices	Management Factors
1. Pre-weaning or lactation handling	Handling practices to ensure adequate piglet colostrum intake
	Age of piglets during weaning (three categories)
	Early water and feed intake during lactation
	Viability of weaned piglets to have a good performance in the post-weaning phase
2. Batch management	Homogeneous batches (same number of farrowings/week ±5%)
	Careful attention to smaller piglets
	Piglets are segregated by weight into different pens
	All-in/all-out system
3. Biosecurity	Foot baths at the weaning room entrance or boot change
	Quarantine for external replacements
	Independent isolation pen or sickbay with special conditions for sick animals
	Proper cleaning, disinfection, and sanitary breaks between different batches
	Independent slurry pit for each post-weaning room
	Change in clothing and boots for visitors
	The distance to other farms or roads is greater than 2 km
	Adequate rodent control program
4. Water quality and access (water management)	Adequate water flow (drinkers: minimum 1l/min)
	Chlorinated water or water with potabilization treatment
	Periodic pipe cleaning (biofilm removal)
	Cleaning water tanks as part of the all-in all-out process in each post-weaning department or room
	Annual microbiological water analysis
	Acidification of water in the first days of post-weaning
	Correct number of drinkers (≥1 drinker per 10 piglets)
5. Feed management	Morning weaning to reduce piglet stress and facilitate feed intake in the first hours
	Adequate feeders for early feed intake after weaning (for instance, plate feeders)
	Appropriate feeder design and space per pig
	Gruel feeding during weaning (to create a liquid feed)
	Rehydrating sources for piglets during weaning
6. Health program	Swine dysentery negative
	PRRS status of breeding sows’ herd (negative or positive with piglet vaccination)
	Monitoring and control of causes of death
	Adequate adaptation program for gilts
	Piglet vaccination against Mycoplasma
	Piglet vaccination against Circovirus
7. Farm stockmen training	Clear instructions and objectives are provided
	There is a performance-based incentive policy
	Periodic training activities are conducted
	The stockmen regularly receive information on weaning results and assess these
8. Post-weaning room temperature management	Adequate thermal insulation
	Type of heating systems
	There are temperature regulators (three possible categories are considered) and records
9. Post-weaning room ventilation management	Type of ventilation
	A ventilation control system exists
	Minimum (5–10%) ventilation of air is ensured or programmed
	Homogeneous air distribution exists
10. Floor type and density in post-weaning rooms	Percentage of slat surface and bedding
	Material of slat floor (three possible categories are considered)
	Correct densities (≥0.1 m^2^/10 kg live weight)
	There is an available area of solid floor without roughness

**Table 2:** Descriptive statistics of the farms’ productive and health parameters (N = 23).

Productive and Health Traits	Mean	SD	Min	Max	Percentiles		
					25	50	75
Number of reproductive sows	704.30	364.42	187.00	1502.00	404.00	690.00	862.00
Number of post-weaning rooms	10.52	5.48	3.00	26.00	6.00	9.00	14.00
Number of piglets weaned per sow per year	24.60	3.20	15.32	28.72	22.61	25.38	26.31
Pre-weaning piglet mortality rate (%)	10.28	4.37	0.78	18.27	6.78	9.39	14.49
Piglet weight during weaning (kg)	5.67	0.18	5.47	6.06	5.51	5.60	5.77
Cost of weaned piglet (EUR)	24.69	3.60	21.43	38.19	22.37	23.66	26.37
Average daily gain during the post-weaning phase (g/day)	310.78	38.71	257.80	395.01	279.51	310.69	342.77
Feed conversion ratio during the post-weaning phase	1.76	0.16	1.20	1.95	1.67	1.78	1.89
Medication costs per piglet during the post-weaning phase (EUR)	5.69	1.44	4.28	10.79	4.77	5.27	6.17
Post-weaning piglet mortality rate (%)	3.46	1.47	1.35	7.49	2.60	3.00	4.05
Total cost per piglet during the post-weaning phase (EUR)	45.14	4.70	39.69	56.94	41.90	44.10	46.74
Cost per kg of live weight of piglet during the post-weaning phase (EUR)	2.33	0.31	2.05	3.07	2.12	2.22	2.40

**Table 3:** Descriptive statistics of veterinary management and hygiene indices scores obtained with the quick scan handling and hygiene calculator (N = 23).

	Mean	SD	Min	Max	Percentiles		
					25	50	75
Pre-weaning handling measures	6.87	1.67	3.75	9.17	5.42	7.08	7.92
Batch management	5.98	2.69	0.00	10.00	5.00	5.00	7.50
Biosecurity	5.11	1.30	1.25	6.25	5.00	5.00	6.25
Water quality and access (water management)	4.81	1.78	2.80	8.40	2.80	4.20	5.60
Feeding management	5.74	1.94	2.00	10.00	4.00	6.00	6.00
Health program	6.28	1.87	0.00	9.00	6.00	7.00	7.00
Farm stockmen training	4.93	2.22	0.00	6.67	3.33	6.67	6.67
Post-weaning room temperature management	4.08	1.89	1.19	7.50	3.33	3.33	5.83
Post-weaning room ventilation management	4.32	2.59	1.00	9.27	2.75	3.00	6.83
Floor type and density in post-weaning rooms	8.03	0.73	6.02	8.75	7.50	8.04	8.75
Farm overall score for the quick scan handling and hygiene calculator	56.12	10.12	37.65	76.76	49.73	54.88	64.36

**Table 4:** Pearson’s correlation coefficients ^1^ between the indices of the quick scan handling and hygiene calculator and the productive and health parameters of the farms (N = 23).

	Number of Piglets Weaned Per Sow per Year	Cost of Weaned Piglet	ADG ^2^ during Post-Weaning	FCR ^2^ during Post-Weaning	Medication Costs per Piglet during Post-Weaning	Post-Weaning Piglet Mortality Rate	Total Cost of Piglet during Post-Weaning	Cost per kg of Live Weight of Piglet during Post-Weaning
Pre-weaning handling measures	0.531 **	−0.396	0.036	−0.322	−0.473 *	−0.490 *	−0.515 *	−0.561 **
Batch management	0.679 ***	−0.494 *	0.14	−0.283	−0.536 **	−0.477 *	−0.656 ***	−0.557 **
Biosecurity	0.547 **	−0.134	0.065	−0.453 *	−0.619 **	−0.455 *	−0.473 *	−0.273
Water quality and access (water management)	0.157	−0.204	−0.168	−0.124	0.141	0.309	−0.179	0.024
Feeding management	0.19	−0.107	0.254	−0.26	−0.049	−0.11	−0.177	−0.071
Health program	−0.199	0.295	0.308	−0.325	0.089	0.108	0.213	0.175
Farm stockmen training	0.389	0.012	0.344	−0.137	−0.432 *	−0.436 *	−0.147	−0.169
Post-weaning room temperature management	0.393	−0.055	−0.167	−0.195	−0.097	−0.239	−0.21	0.122
Post-weaning room ventilation management	0.301	0.078	−0.304	−0.218	−0.162	−0.286	−0.152	0.095
Floor type and density in post-weaning rooms	−0.085	0.236	−0.173	−0.063	0.167	−0.058	0.248	0.219
Farm overall score for the quick scan handling and hygiene calculator	0.592 **	−0.182	0.086	−0.442 *	−0.414 *	−0.437 *	−0.435 *	−0.228

^1^ Significance of the correlation coefficients (two-tailed test): *, **, and *** significant at *p* ≤ 0.05, *p* ≤ 0.01, and *p* ≤ 0.001, respectively. ^2^ ADG = average daily gain; FCR = feed conversion ratio.

**Table 5:** Means (standard deviation) of productive parameters and the overall score of the quick scan handling and hygiene calculator by farm quartiles based on annual productivity (N = 23).

	Q1. 25% of Farms with the Highest Annual Productivity	Q2 and Q3 (50% of the Farms)	Q4. 25% of Farms with the Lowest Annual Productivity	Total
Number of piglets weaned per sow per year	27.73 ^a^ (0.88)	25.29 ^b^ (0.74)	20.20 ^c^ (2.70)	24.60 (3.20)
Farm overall score for the quick scan handling and hygiene calculator	68.23 ^a^ (8.22)	52.83 ^b^ (5.22)	50.04 ^b^ (9.16)	56.12 (10.12)
Mean number of reproductive sows	714.83 (512.55)	808.00 (318.39)	503.67 (216.32)	704.30 (364.42)
Pre-weaning piglet mortality rate	7.42 ^a^ (3.74)	9.54 ^a^ (3.43)	14.51 ^b^ (3.73)	10.28 (4.37)
Piglet weight during weaning (kg)	5.63 (0.16)	5.65 (0.17)	5.74 (0.24)	5.67 (0.18)
Cost of weaned piglet (EUR)	22.31 ^a^ (1.24)	24.38 ^ab^ (1.63)	27.64 ^b^ (5.73)	24.69 (3.60)
ADG ^1^ during the post-weaning phase (g/d)	322.84 (44.70)	300.16 (32.56)	318.19 (44.58)	310.78 (38.71)
FCR ^2^ during the post-weaning phase	1.70 (0.06)	1.80 (0.11)	1.73 (0.28)	1.76 (0.16)
Medication costs per piglet during the post-weaning phase (EUR)	5.16 ^a^ (0.53)	5.13 ^a^ (0.56)	7.25 ^b^ (2.06)	5.69 (1.44)
Post-weaning piglet mortality rate	2.91 ^a^ (0.79)	2.96 ^a^ (0.89)	4.93 ^b^ (1.94)	3.46 (1.47)
Total cost per piglet during the post-weaning phase	42.12 ^a^ (2.49)	44.38 ^a^ (2.31)	49.56 ^b^ (6.69)	45.14 (4.70)
Cost per kg of live weight of the piglet during the post-weaning phase	2.14 ^a^ (0.11)	2.31 ^ab^ (0.28)	2.57 ^b^ (0.38)	2.33 (0.31)

^1^ ADG = average daily gain; ^2^ FCR = feed conversion ratio. ^a–c^ Values within a row with different superscripts indicate significant differences between groups (*p* < 0.05).

**Table 6:** Means (standard deviation) of productive parameters and the overall score of the quick scan handling and hygiene calculator by farm quartiles based on the cost of veterinary medications per weaner (N = 23).

	Q1. 25% of Farms with the Highest Cost of Medications per Piglet during Post-Weaning	Q2 and Q3 (50% of the Farms)	Q4. 25% of Farms with the Lowest Cost of Medications per Piglet during Post-Weaning	Total
Medication costs per piglet during the post-weaning phase (EUR)	7.54 ^a^ (1.72)	5.29 ^b^ (0.27)	4.58 ^b^ (0.19)	5.69 (1.44)
Farm overall score for the quick scan handling and hygiene calculator	49.90 (9.07)	59.64 (10.85)	55.88 (7.67)	56.12 (10.12)
Mean number of reproductive sows	565.33 (263.65)	793.00 (434.40)	680.67 (310.28)	704.30 (364.42)
Number of piglets weaned per sow per year	20.78 ^a^ (3.42)	26.15 ^b^ (1.57)	25.59 ^b^ (2.16)	24.60 (3.20)
Pre-weaning piglet mortality rate	13.03 (3.70)	8.60 (3.74)	10.61 (5.19)	10.28 (4.37)
Piglet weight during weaning (kg)	5.72 (0.26)	5.68 (0.16)	5.61 (0.15)	5.67 (0.18)
Cost of weaned piglet (EUR)	28.02 ^a^ (5.42)	23.78 ^b^ (2.03)	23.02 ^b^ (1.05)	24.69 (3.60)
^1^ ADG during the post-weaning phase (g/day)	312.73 (48.45)	313.27 (43.45)	304.26 (20.01)	310.78 (38.71)
^2^ FCR during the post-weaning phase	1.72 (0.27)	1.76 (0.10)	1.79 (0.14)	1.76 (0.16)
Post-weaning piglet mortality rate	4.74 ^a^ (2.13)	3.07 ^b^ (0.90)	2.91 ^b^ (0.83)	3.46 (1.47)
Total cost per piglet during the post-weaning phase	50.31 ^a^ (5.83)	43.81 ^b^ (2.72)	42.41 ^b^ (1.92)	45.14 (4.70)
Cost per kilogram of live weight of the piglet during the post-weaning phase	2.59 ^a^ (0.36)	2.30 ^ab^ (0.29)	2.14 ^b^ (0.08)	2.33 (0.31)

^1^ ADG = average daily gain; ^2^ FCR = feed conversion ratio. ^a–b^ Values within a row with different superscripts indicate significant differences between groups (*p* < 0.05).

## Data Availability

These data are not deposited in an official repository due to confidentiality constraints. However, a portion of these data may be available upon request from Santos Sanz-Fernández (v22safes@uco.es).

## Supplementary Materials

The following supporting information can be downloaded at https://www.mdpi.com/article/10.3390/ani13223508/s1, Table S1: Scores of each index and their associated management factors in the quick scan handling and hygiene calculator.

## References

[1] Contreras J.M., Calderón Á., López J.Á. (2012). La Nutrición Del Lechón En Relación Con Los Puntos Críticos En El Destete. Anaporc.

[2] Prieto Barona S., Díaz Gaona C., Sánchez Rodríguez M., Rodríguez Estévez V. (2017). El manejo de los lechones destetados. Ganadería.

[3] Pluske J.R., Pethick D.W., Hopwood D.E., Hampson D.J. (2002). Nutritional Influences on Some Major Enteric Bacterial Diseases of Pig. Nutr. Res. Rev..

[4] Pluske J.R., Turpin D.L., Kim J.-C. (2018). Gastrointestinal Tract (Gut) Health in the Young Pig. Anim. Nutr..

[5] Diana A., Boyle L.A., Leonard F.C., Carroll C., Sheehan E., Murphy D., Manzanilla E.G. (2019). Removing Prophylactic Antibiotics from Pig Feed: How Does It Affect Their Performance and Health?. BMC Vet. Res..

[6] Pluske J.R., Miller D.W., Sterndale S.O., Turpin D.L., Pluske J.R., Miller D.W., Sterndale S.O., Turpin D.L. (2019). Associations between Gastrointestinal-Tract Function and the Stress Response after Weaning in Pigs. Anim. Prod. Sci..

[7] Magallón E., García A., Bautista R., Alonso B., Cano J.I., Almenara S., Prieto P., Magallón P., Ortiz E. (2017). Manejo y Gestión del Posdestete. El Lechón Destetado—Grupo Asís.

[8] Walsh C., Fanning S. (2008). Antimicrobial Resistance in Foodborne Pathogens—A Cause for Concern?. Curr. Drug Targets.

[9] Murphy D., Ricci A., Auce Z., Beechinor J.G., Bergendahl H., Breathnach R., Bureš J., Silva J.P.D.D., Hederová J., Hekman P. (2017). EMA and EFSA Joint Scientific Opinion on Measures to Reduce the Need to Use Antimicrobial Agents in Animal Husbandry in the European Union, and the Resulting Impacts on Food Safety (RONAFA). EFSA J..

[10] Temple D., Dalmau A., Ruiz de la Torre J.L., Manteca X., Velarde A. (2011). Application of the Welfare Quality^®^ Protocol to Assess Growing Pigs Kept under Intensive Conditions in Spain. J. Vet. Behav..

[11] Agostini P.S., Gasó J.G., Manzanilla E.G., Silva C.A.D., de Beorlegui C.B. (2013). Descriptive Study of Production Factors Affecting Performance Traits in Growing-Finishing Pigs in Spain. Span. J. Agric. Res..

[12] Backhans A., Sjölund M., Lindberg A., Emanuelson U. (2015). Biosecurity Level and Health Management Practices in 60 Swedish Farrow-to-Finish Herds. Acta Vet. Scand..

[13] Black J.L., Giles L.R., Wynn P.C., Knowles A.G., Kerr C.A., Jones M.R., Strom A.D., Gallagher N.L., Eamens G.J. (2001). Factors Limiting the Performance of Growing Pigs in Commercial Environments. Manipulating pig production VIII. Proceedings of the Eighth Biennial Conference of the Australasian Pig Science Association (APSA), Adelaide, Australia, 25–28 November, 2001.

[14] Da Silva C.A., Agostini P.D.S., Dias C.P., Callegari M.A., dos Santos R.d.K.S., Novais A.K., Pierozan C.R., Gasó J.G. (2017). Characterization and Influence of Production Factors on Growing and Finishing Pig Farms in Brazilian Cooperatives. R. Bras. Zootec..

[15] Laine T.M., Lyytikäinen T., Yliaho M., Anttila M. (2008). Risk Factors for Post-Weaning Diarrhoea on Piglet Producing Farms in Finland. Acta Vet. Scand..

[16] Postma M., Stärk K.D.C., Sjölund M., Backhans A., Beilage E.G., Lösken S., Belloc C., Collineau L., Iten D., Visschers V. (2015). Alternatives to the Use of Antimicrobial Agents in Pig Production: A Multi-Country Expert-Ranking of Perceived Effectiveness, Feasibility and Return on Investment. Prev. Vet. Med..

[17] Gleeson B.L., Collins A.M., Gleeson B.L., Collins A.M. (2015). Under What Conditions Is It Possible to Produce Pigs without Using Antimicrobials?. Anim. Prod. Sci..

[18] Rojo-Gimeno C., Postma M., Dewulf J., Hogeveen H., Lauwers L., Wauters E. (2016). Farm-Economic Analysis of Reducing Antimicrobial Use Whilst Adopting Improved Management Strategies on Farrow-to-Finish Pig Farms. Prev. Vet. Med..

[19] Raasch S., Collineau L., Postma M., Backhans A., Sjölund M., Belloc C., Emanuelson U., Beilage E.G., Stärk K., Dewulf J. (2020). Effectiveness of Alternative Measures to Reduce Antimicrobial Usage in Pig Production in Four European Countries. Porc. Health Manag..

[20] Lynegaard J.C., Kjeldsen N.J., Bache J.K., Weber N.R., Hansen C.F., Nielsen J.P., Amdi C. (2021). Low Protein Diets without Medicinal Zinc Oxide for Weaned Pigs Reduced Diarrhea Treatments and Average Daily Gain. Animal.

[21] Waluszewski A., Cinti A., Perna A. (2021). Antibiotics in Pig Meat Production: Restrictions as the Odd Case and Overuse as Normality? Experiences from Sweden and Italy. Humanit. Soc. Sci. Commun..

[22] Carr J. (2004). Estándares de La Producción Porcina: Manual Técnico.

[23] da Agostini P.S., Manzanilla E.G., de Blas C., Fahey A.G., da Silva C.A., Gasa J. (2015). Managing Variability in Decision Making in Swine Growing-Finishing Units. Ir. Vet. J..

[24] Faucitano L., Schaefer A.L. (2008). Welfare of Pigs: From Birth to Slaughter.

[25] Velarde A., Geers R. (2007). On Farm Monitoring of Pig Welfare.

[26] Sanz-Fernández S., Díaz Gaona C., Casas-Rosal J.C., Alos N., Tusell L., Quintanilla R., Rodríguez-Estévez V. (2023). Pre-Weaning Piglet Survival on Commercial Farms. J. Anim. Sci..

[27] Andersen I.L., Tajet G.M., Haukvik I.A., Kongsrud S., Bøe K.E. (2007). Relationship between Postnatal Piglet Mortality, Environmental Factors and Management around Farrowing in Herds with Loose-Housed, Lactating Sows. Acta Agric. Scand. Sect. A—Anim. Sci..

[28] Koketsu Y., Iida R. (2020). Farm Data Analysis for Lifetime Performance Components of Sows and Their Predictors in Breeding Herds. Porc. Health Manag..

[29] Moreira L.P., Menegat M.B., Barros G.P., Bernardi M.L., Wentz I., Bortolozzo F.P. (2017). Effects of Colostrum, and Protein and Energy Supplementation on Survival and Performance of Low-Birth-Weight Piglets. Livest. Sci..

[30] Soraci A.L., Amanto F., Tapia M.O., de la Torre E., Toutain P.-L. (2014). Exposure Variability of Fosfomycin Administered to Pigs in Food or Water: Impact of Social Rank. Res. Vet. Sci..

[31] Camp Montoro J., Pessoa J., Solà-Oriol D., Muns R., Gasa J., Manzanilla E.G. (2022). Effect of Phase Feeding, Space Allowance and Mixing on Productive Performance of Grower-Finisher Pigs. Animals.

[32] López-Vergé S., Solà-Oriol, Gasa J. Strategies to Control Piglet Weight Variability in the Nursery (1/2): Farrowing, Segregation of Animals, Environmental Comfort. https://www.pig333.com/articles/strategies-to-control-piglet-weight-variability-in-the-nursery-1-2_10964/.

[33] Bottoms K., Poljak Z., Dewey C., Deardon R., Holtkamp D., Friendship R. (2013). Evaluation of External Biosecurity Practices on Southern Ontario Sow Farms. Prev. Vet. Med..

[34] Laanen M., Persoons D., Ribbens S., de Jong E., Callens B., Strubbe M., Maes D., Dewulf J. (2013). Relationship between Biosecurity and Production/Antimicrobial Treatment Characteristics in Pig Herds. Vet. J..

[35] Postma M., Backhans A., Collineau L., Loesken S., Sjölund M., Belloc C., Emanuelson U., grosse Beilage E., Nielsen E.O., Stärk K.D.C. (2016). Evaluation of the Relationship between the Biosecurity Status, Production Parameters, Herd Characteristics and Antimicrobial Usage in Farrow-to-Finish Pig Production in Four EU Countries. Porc. Health Manag..

[36] Kummer R., Gonçalves M.A.D., Lippke R.T. (2009). Fatores que influenciam o desempenho dos leitões na fase de creche. Acta Sci. Vet..

[37] Rodríguez Estévez V., Díaz Gaona C., Arce C., Sánchez Rodríguez M. (2016). Los Cuidados del lechón en el Destete. Albéitar Publicación Vet. Indep..

[38] Madec F., Bridoux N., Bounaix S., Jestin A. (1998). Measurement of Digestive Disorders in the Piglet at Weaning and Related Risk Factors. Prev. Vet. Med..

[39] Rhouma M., Fairbrother J.M., Beaudry F., Letellier A. (2017). Post Weaning Diarrhea in Pigs: Risk Factors and Non-Colistin-Based Control Strategies. Acta Vet. Scand..

[40] Fornós M., Sanz-Fernández S., Jiménez-Moreno E., Carrión D., Gasa J., Rodríguez-Estévez V. (2022). The Feeding Behaviour Habits of Growing-Finishing Pigs and Its Effects on Growth Performance and Carcass Quality: A Review. Animals.

[41] O’Connell N.E., Beattie V.E., Weatherup R.N. (2002). Influence of Feeder Type on the Performance and Behaviour of Weaned Pigs. Livest. Prod. Sci..

[42] Kemper N. (2020). Optimising Pig Welfare at the Weaning and Nursery Stage. Understanding the Behaviour and Improving the Welfare of Pigs.

[43] Magallón E., García A., Bautista R., Alonso B., Cano J.I., Prieto P., Magallón P. (2015). Manejo y Gestión de Maternidades Porcinas II. La Lactación- Grupo Asís Store.

[44] Spoolder H.A.M., Ruis M.A.W. (2015). Improving Farm Animal Productivity and Welfare, by Increasing Skills and Knowledge of Stock People. Environment and Welfare—Proceedings of International Symposium.

[45] Kil D.Y., Stein H.H. (2010). Board Invited Review: Management and Feeding Strategies to Ameliorate the Impact of Removing Antibiotic Growth Promoters from Diets Fed to Weanling Pigs. Can. J. Anim. Sci..

[46] Chantziaras I., De Meyer D., Vrielinck L., Van Limbergen T., Pineiro C., Dewulf J., Kyriazakis I., Maes D. (2020). Environment-, Health-, Performance- and Welfare-Related Parameters in Pig Barns with Natural and Mechanical Ventilation. Prev. Vet. Med..

[47] Ramirez B.C., Hayes M.D., Condotta I.C.F.S., Leonard S.M. (2022). Impact of Housing Environment and Management on Pre-/Post-Weaning Piglet Productivity. J. Anim. Sci..

[48] Moreno R., Buxadé C. (1999). Instalaciones para lechones y cerdos de cebo. Mundo Ganadero.

[49] Ye Z., Zhang G., Seo I.-H., Kai P., Saha C.K., Wang C., Li B. (2009). Airflow Characteristics at the Surface of Manure in a Storage Pit Affected by Ventilation Rate, Floor Slat Opening, and Headspace Height. Biosyst. Eng..

[50] Morsing S., Strøm J.S., Zhang G., Kai P. (2008). Scale Model Experiments to Determine the Effects of Internal Airflow and Floor Design on Gaseous Emissions from Animal Houses. Biosyst. Eng..

[51] Kim J., Lee I., Lee S., Park S., Jeong D., Choi Y., Decano-Valentin C., Yeo U. (2022). Development of an Air-Recirculated Ventilation System for a Piglet House, Part 1: Analysis of Representative Problems through Field Experiment and Aerodynamic Analysis Using CFD Simulation for Evaluating Applicability of System. Agriculture.

[52] Friendship R.M., Wilson M.R., McMillan I. (1986). Management and Housing Factors Associated with Piglet Preweaning Mortality. Can. Vet. J..

[53] King V.L., Koketsu Y., Reeves D., Xue J., Dial G.D. (1998). Management Factors Associated with Swine Breeding-Herd Productivity in the United States. Prev. Vet. Med..

[54] Pierozan C.R., Callegari M.A., Dias C.P., de Souza K.L., Gasa J., Silva C.A. (2020). da Herd-Level Factors Associated with Piglet Weight at Weaning, Kilograms of Piglets Weaned per Sow per Year and Sow Feed Conversion. Animal.

[55] Koketsu Y., Tani S., Iida R. (2017). Factors for Improving Reproductive Performance of Sows and Herd Productivity in Commercial Breeding Herds. Porc. Health Manag..

[56] Davis R.L., Briggs G.M. (1951). Studies with Antibiotics in Chick and Poult Starting Rations1. Poult. Sci..

[57] Hill D.C., Branion H.D., Slinger S.J., Anderson G.W. (1953). Influence of Environment on the Growth Response of Chicks to Penicillin*. Poult. Sci..

[58] Teillant A., Brower C.H., Laxminarayan R. (2015). Economics of Antibiotic Growth Promoters in Livestock. Annu. Rev. Resour. Econ..

[59] Dritz S.S., Tokach M.D., Goodband R.D., Nelssen J.L. (2002). Effects of Administration of Antimicrobials in Feed on Growth Rate and Feed Efficiency of Pigs in Multisite Production Systems. J. Am. Vet. Med. Assoc..

[60] Miller G.Y., Algozin K.A., McNamara P.E., Bush E.J. (2003). Productivity and Economic Effects of Antibiotics Used for Growth Promotion in U.S. Pork Production. J. Agric. Appl. Econ..

[61] Zimmerman D.R. (1986). Role of Subtherapeutic Levels of Antimicrobials in Pig Production. J. Anim. Sci..

[62] Ardakani Z., Canali M., Aragrande M., Tomassone L., Simoes M., Balzani A., Beber C.L. (2023). Evaluating the Contribution of Antimicrobial Use in Farmed Animals to Global Antimicrobial Resistance in Humans. One Health.

[63] Pitkin A., Otake S., Dee S. (2009). Biosecurity Protocols for the Prevention of Spread of Porcine Reproductive and Respiratory Syndrome Virus.

[64] Pritchard G., Dennis I., Waddilove J. (2005). Biosecurity: Reducing Disease Risks to Pig Breeding Herds. Practice.

[65] Edwards L., Crabb H. (2021). Water Quality and Management in the Australian Pig Industry. Anim. Prod. Sci..

[66] PIC (2009). Wean to Finish Guidelines. Environment: Heat and Humidity Removal..

