# Supplementary material for: Multi-Criteria Evaluation Model of Management for Weaned Piglets and Its Relations with Farm Performance and Veterinary Medicine Consumption
Source: Animals (Basel). 2023 Nov 14;13(22):3508. doi: 10.3390/ani13223508 (PMC10668820; doi:10.3390/ani13223508)
Supplement: Supplementary file 1 [file animals-13-03508-s001.zip › animals-2695641-supplementary.pdf]

Supplementary Material – Table S1.

Table S1. Scores of each index and their associated management factors in the quick scan handling and hygiene calculator

**ID FARM:**	**Please. complete with yes or no**	**Score**	**Notes**
**1. Pre-weaning or lactation handling:**		**10**	
Handling practices to ensure adequate piglet colostrum intake	yes	2.5	
Age of piglets at weaning:	-		
≤ 22 days	no	0	Only one
22-24 days	no	1.25	
≥ 25 days	yes	2.5	
Early water and feed intake during lactation:	-		
Properly cleaned and suitable feeders for piglets	yes	0.833	
Feeding small amounts of prestarter feed multiple times a day to encourage consumption	yes	0.833	
Suitable drinkers for piglets	yes	0.833	
Viability of weaned piglets to have a good performance at the post-weaning phase	yes	2.5	
**2. Batch Management:**		**10**	
Homogeneous batches (same number of farrowings/week = ±5%)	yes	2.5	
Careful attention to smaller piglets	yes	2.5	
Piglets are segregated by weight into different pens	yes	2.5	
All-in/all-out system	yes	2.5	
**3. Biosecurity:**		**10**	
Foot-baths at weaning rooms entrance or boot change	yes	1.25	
Quarantine for external replacements	yes	1.25	
Independent isolation pen or sickbay with special conditions for sick animals	yes	1.25	
Proper cleaning, disinfection and sanitary break between different batches	yes	1.25	
Independent slurry pit for each post-weaning room	yes	1.25	
Change of clothing and boots for visitors	yes	1.25	
The distance to other farms or roads is greater than 2 km	yes	1.25	
Adequate rodent control program	yes	1.25	

**4. Water Quality and Access (water management):**		**10**	
Adequate water flow (drinkers: minimum 1l/min)	yes	1.4	
Chlorinated water or water with potabilization treatment.	yes	1.4	
Periodic pipe cleaning (biofilm removal)	yes	1.4	
Cleaning water tanks as part of the all-in all-out process in each post-weaning department or room	yes	1.4	
Annual microbiological water analysis	yes	1.4	
Acidification of water in the first days of post-weaning	yes	1.4	
Correct number of drinkers (≥1 drinker per 10 piglets)	yes	1.4	
**5. Feed management:**		**10**	
Morning weaning to reduce piglet stress and facilitate feed intake in the first hours	yes	2	
Adequate feeders for early feed intake after weaning (for instance, plate feeders)	yes	2	
Appropriate feeders design and space per pig	yes	2	
Gruel feeding at weaning (to create a liquid feed)	yes	2	
Rehydrating sources for piglets at weaning	yes	2	
**6. Health Program:**		**10**	
Swine dysentery negative	yes	1	
PRRS status of breeding sows' herd	-		
PRRS negative	yes	2	Only one
PRRS positive with piglet vaccination	no	0.5	
Monitoring and control of causes of death	yes	1	
Adequate adaptation program for gilts	yes	3	
Piglet vaccination against Mycoplasma	yes	1.5	
Piglet vaccination against Circovirus	yes	1.5	
**7. Farm stockmen training:**		**10**	
Clear instructions and objectives are provided	yes	3.33	
There is a performance-based incentive policy	yes	1.67	
Periodic training activities are conducted	yes	1.67	
The stockmen regularly receive information of weaning results and assess these	yes	3.33	
**8. Post-weaning rooms temperature management:**		**10**	
Adequate thermal insulation	yes	1.67	
Type of heating systems	-		

Localized heating (heats the area occupied by the animal: thermal floor or plate)	yes	1.67	
Ambient heating (heats the volume of the room: convection or thermal radiation)	yes	1.67	
There are temperature regulators and records	-		
Temperature regulators are available	yes	1.67	
Temperature records exist	yes	1.67	
Temperature programming is implemented	yes	1.67	
**9. Post-weaning rooms ventilation management:**		**10**	
Type of ventilation	-		
Natural ventilation	yes	1	
Forced ventilation	yes	2	
Ventilation control system exists	yes	2	
Minimum (5-10%) ventilation of air is ensured or programmed	yes	3	
Homogeneous air distribution exists	yes	2	
**10. Floor type and density in post-weaning rooms:**		**10**	
Percentage of slats surface and bedding	-		
Surface ≥50% slats	yes	1.25	
Surface <50% slats without bedding	yes	0	
Surface <50% slats with bedding	yes	1.25	
Material of slat floor	-		
Plastic slat floor	yes	1.25	Only one
Concrete slat floor	no	0	
Metal slat floor	no	0.625	
Correct densities (≥0.1 m2/10 kg live weight)	yes	5	
There is an available area of solid floor without roughness	yes	1.25	
**Maximum overall score for the quick scan handling and hygiene calculator**		**100**	
